# Some Physical Defects in Children and Their Effect upon Development

**Published:** 1907-09

**Authors:** Franklin W. Bock

**Affiliations:** Rochester, N. Y.; One of the Oto-Laryngologists of the Rochester Public Health Association


### Some Physical Defects in Children and Their Effect Upon Development.

### A Plea for the Systematic Examination and Treatment of Children.

BY FRANKLIN W. BOCK, M. D., Rochester, N. Y.
One of the Oto-Laryngologists of the Rochester Public Health Association.

HERBERT SPENCER said "to be a good animal is the first requisite to success in life, and to be a nation of good animals is the first condition to national prosperity." That sufficient attention has not been given to the preparation of the young human animal for its life work cannot be denied, but fortunately the many diverse opinions which have held sway so long as to the effect of various physical defects upon the development of children, are gradually becoming reconciled and more and more we are coming to believe and to know, that many of the functional and physical defects of which we used to think little and in fact often were ignorant of their existence if not the actual cause,

1. Read before the Monroe County Medical Society, May 12, 1907.

are at least very potent factors in retarding healthy development, physically, morally, mentally and spiritually.

Take a child with poor accommodation, who with difficulty breathes through the nose on account of some nasal disease or deformity, with a nasopharynx filled with dripping adenoids, with enlarged and diseased tonsils, with bad teeth, with a reduction in hearing or sight or with any of the other functional and physical defects which we find in children, and is it not unreasonable to expect that child to do as good or as much work as one who is comparatively perfect physically? Is it not unreasonable to expect such a child to attain to any sort of perfection in mental or moral development?

What do you do if your head aches, or your teeth ache, or you can't hear, or your eyes swim? First, you make yourself miserable and then proceed to make every one else miserable. Then someone with more than ordinary good sense says, "had you not better see a doctor?" Taking the hint you go and find you have eye strain, or a bad nasal condition, or bad teeth, or reduced hearing or sight, or some other remediable condition. You get relieved, hence feel better and act better, and when you tell about it afterward you pat yourself on the back for a long suffering martyr.

Did it ever occur to you that that morose, petulant boy of yours perhaps had one or more of the same conditions which made a bear of you? That the one who doesn't seem to get along well at school, may not be able to see without strain, or may only hear half that the teacher says? Did it ever occur to you that a child laboring under one or more of these physical handicaps had a pretty good excuse for being backward or fussy or even bad. Every physician realises how difficult it is often for an adult, with years of training behind him, to interpret the symptoms from which he suffers, due often to some seemingly simple little physical disability. Yet many of our children are being punished because they are bad, and overworked when they are backward, while every day they are tearing their little hearts out because they cannot interpret the symptom complex which is depressing them mentally, or stirring up the evil spirit within them.

We have recognised that much of the faulty social development of the child is due to bad home conditions and we have established social settlements with manual training classes and the like, where they are taught many things which go to make home pleasant; we have recognised that faulty moral development is often due to bad conditions of recreation or no recreation at all, and we have established play grounds where their ideals of recreation are led into legitimate healthy channels. Is there anyone who does not now recognise the good these institutions are doing for the individual and the community.

The lay members of the community have done much to improve the social and moral conditions of the children, but what have we as a profession done to improve their physical and mental condition? Have we a family physician in Rochester who regularly examines the children of his patrons, seeking functional and physical defects with a view of improving their mental and physical development and to prevent disease? Is there a physician in Rochester who is paid a regular amount by the year to keep a family well? This may seem like reiterating an oft repeated dream of the past, but is it unreasonable to ask the physicians of Rochester to give a little more thought to this matter of the systematic examination and treatment of children with a view of improving the race?

I am not prepared to say how and by whom this work should be done, but at any rate there is so much *to be* done that whoever takes it up, the active cooperation of every physician in the city will be needed in one way or another. An educated public opinion must first be created and who but the physicians should be the educators, the creators? At present this work is being carried on by the Rochester Public Health Association at its clinics at 32 S. Washington street, and for the sake of any who may not know, I will say that this organisation is not a part of the City Health Department, but is supported by voluntary subscription only. It was founded in 1898 on the occasion of the golden wedding of Dr. E. M. Moore, Sr., and since then it has been endeavoring by means of lectures, literature, by demonstration, and actual clinical work to disseminate knowledge as to ways and means of preventing disease as well as improve individual and the public health.

Its first clinical work was done with tuberculosis patients in the city, and a trained nurse still gives a large part of her time to visiting such patients and instructing them in personal hygiene as well as how to prevent the spread of the disease. It maintains a dental, an eye, and an ear, nose and throat clinic. The work in these clinics has gradually brought them to the position of being almost purely children's clinics. We feel that our best work is with them and if by elevating the physical, mental and moral standards of the children of Rochester, we impress upon the people the necessity of our existence, we shall feel satisfied.

It is because of my interest in this work with the children and my association in the nose and throat clinic that I wish to present today certain observations which we have made during the past few months. It must be remembered that in no case has a complete general examination been made, and the conditions noted which are not directly related to ear, nose and throat work were discovered because of symptoms which pointed to them. I always note the condition of the teeth, and, when necessary, refer cases to the dental clinic for treatment, and almost without exception I have found it necessary to do so. I believe that among the school children of Rochester there is more than enough necessary dental work to keep a dozen dentists busy for a year. Lately I have also taken note of eye conditions and where there was the least suspicion of defect have referred them to the eye clinic for examination.

Again, these cases are not selected. Perhaps ten or twelve have been sent to us especially for ear, nose or throat examination. Of the remainder about a third were sent for eye or dental work, and I have examined them also. I have gone out into the highways and byways and invited the remainder to come in. In other words I have examined every child I could get hold of. With few exceptions the parents have been ignorant of any trouble whatever.

From January 1 to May 1, we have examined 164 children, the average age being ten years. The following conditions were found:

145 had enlarged tonsils.
9 had small tonsils.
136 had adenoids.
1 had acute tonsilitis.
3 had frequent tonsilitis.
46 knew they often had earache.
5 knew they had earache and deafness with every cold.
1 had earache and discharge with every cold.
18 knew they had had discharging ears at some time.
13 had impacted wax.
29 had tympanic membranes which indicated a previous acute attack.
1 had eczema of external ear.
1 had chronic purulent otitis media.
45 had reduced hearing from 2-3 down to contact.
32 had less than ½ normal hearing.
3 were deaf from scarlet fever.
2 had noises in their ears.
1 had mastoid swelling with every cold.
1 had had an old mastoid abscess.
1 had acute mastoiditis.
22 had enlarged post-cervical glands.
2 had rheumatic sore throat.
6 had chronic laryngitis.
5 had chronic cough.
2 had whooping cough.

1 had asthma.
1 had shortness of breath.
6 had difficult nasal respiration.
1 had contracted nostrils.
1 had a broken nose, cartilages displaced.
3 had deflected septums. In two of these one side was closed.
1 had frequent nose bleed.
2 had bad nasal discharge.
7 had bad postnasal discharge.
7 had chronic rhinitis.
3 had chronic purulent rhinitis.
1 had atrophic rhinitis.
82 had bad teeth; these do not include about 50 who came to me from the dental clinic.
6 only had good teeth.
1 had a gum boil.
1 stutters.
1 had phimosis, 3½ years old.
1 had goiter.
1 had goiter which became tender with every cold.
1 had goiter with slight exophthalmos and great nervousness.
1 had congenital atrophy of one optic nerve,—reduced vision in the other eye.
1 had traumatic blindness of one eye,—normal vision in the other eye.

A few combinations found.

1 boy had a tubercular thumb joint, a tubercular right lung, enlarged tonsils, a deflected septum, and bad teeth.
1 girl had traumatic blindness in one eye; marked increasing deafness, enlarged tonsils, adenoids and bad teeth.
1 had phimosis, enlarged tonsils, adenoids and bad teeth.
1 had corneal scars both eyes, gonorrheal ophthalmia; reduced sight, tonsils and adenoids, enlarged postcervical and submaxillary glands and bad teeth. Mother and brother dead of tuberculosis and one brother with it now. 12 years old, not in school for a year.

Of these cases practically 100% had some physical defect. I do not remember one child who could be considered as in first class condition. Perhaps fifty per cent. of these cases came from the north side of the city, the rest are scattered pretty evenly over the other sections so they represent a fair type of the average child in Rochester. But, for the sake of fairness, suppose it represents the condition of only fifty per cent. of our children, I think you will agree with me that here is much food for conscientious thought. It means that fifteen thousand children in Rochester are suffering from one or more remediable physical defects.

That persons with reduced hearing or eye sight, are necessarily barred from certain industries, although otherwise capable; that children thus affected are more liable to accident; that children with enlarged diseased tonsils and adenoids and bad teeth are more susceptible to infectious diseases, and that they are a most potent factor in the spread of such infection; that children of working age, because of their greater susceptibility, are frequently ill and have to stop work and thus retard the wheels of industry—these all are facts which would have to be taken into account were we to consider this matter from a politico-economic standpoint, but of greater moment at this time is the question, what influences have these defects upon the mental and moral development of the child?

I doubt if anyone could for a moment believe that one or more of these defects could result in good to the economy, so there only remains for us to decide, is their effect negative or is it bad? We have many children whose general health seems not to be affected deleteriously, of whom the parents may say "Why she hasn't had a sick day in her life." Even in these cases it is my opinion that the effect is not negative but actually bad. I hazard this statement because of the simple but quite convincing experience that I have yet to see the child, in whom the effects have seemingly been negative, who has not been better after operation than before, and I have had many such experiences. I believe that functional and physical defects always have more or less bad effect upon the moral and mental development of the child; that however well or bright or good he may be with these defects, he will be healthier, brighter and better without them. We are yet but in the early dawn of this movement but the results are so convincing that I believe honestly that the neglect of this duty is adding daily to our proportion of bad citizens.

Dr. Cronin, chief inspector of schools of New York, says: they have examined, up to September 1906, 99,000 of the 600,000 children in New York city; that 66% of those examined showed need of medical attention; that 95% of backward children have some physical defect; that their experience had been that 95% of these improve in conduct and efficiency in study after operation or the proper fitting of glasses.

Dr. Charles Bernstein, Superintendent of the State Custodial Asylum at Rome, said in a letter to me a few days ago: "Our experience with the feeble-minded in this institution has convinced us that not sufficient attention is paid to the physical condition of children, especially as regards their eyes, noses, condition of hearing, and particularly the condition of the intestinal tract. As striking examples we can cite the following cases: A boy with favus of the scalp was very feeble-minded, and as a result of curing this disease of the scalp his mind apparently entirely cleared up. The child was taken home and appears as bright as the rest of the children. Another boy, as the result of intestinal disturbances and faulty digestion and nutrition, was entirely cured of his feeble-minded condition by clearing up his intestinal tract. A number of cases of feeble-mindedness, the result of mouth breathing, because of nasal polypus, in which the mental condition decidedly improved as the result of the removal of these obstructions. The driveling idiot type has been eliminated to a large extent by dental treatment, with a consequent improvement in mental condition. Also a number of cases where, as the result of operation for phymosis, the mental condition was very markedly altered for good. Dr. Evans of the University of Pennsylvania says: "Eighty per cent. of the truants from school are suffering from defective eyesight." Dr. Allport of Chicago says: "If the direct cause of criminality and pauperism could be accurately ascertained, I venture the opinion that the prevailing etiological factors would be physical defectiveness and social surroundings."

Now to come nearer home. What is the effect upon Rochester children? I took a list of 46 children which I had examined in four different schools and obtained the standing of each:

4 were excellent.
9 were good.
14 were fair.
10 were poor.
9 were very poor.

The standing in each case, with one or two exceptions, is directly related to the severity of the nose, ear and throat conditions. One child with ¼ hearing is in the good class. The five others with reduced hearing are in the poor or very poor class. The difference between the age of backward pupils and those in better standing is seen to increase steadily from the first to the eighth grade. This is the natural result of the discouragement which comes to a backward child, when he sees his more fortunate companions steadily going ahead of him, and of the increasing lack of attention which the backward pupil gets even in the best of classes.

I have lately examined a number of children in several of our schools who are known to be very backward. Every one of these needs work done very badly in nose, throat or mouth, and some need glasses. One little eight-year old boy is a very bad case of adenoids and tonsils. This boy at three and a half could count to twenty and knew all his letters. Today he can only count three and knows only A and B of his letters. I have the promise of this boy's parents to allow me to operate upon him, and also the permission of several other of these children, so that sometime in the future, I hope to give the results of this work with these very backward children. One boy I have operated upon, but it is too early to give any results except to say that when I examined him he had both ears filled with very hard wax. It took several days to remove it all. He could hear a watch at six inches with one ear and not at all with the other. After removing the wax he could hear the watch at five feet, and the next time he saw his teacher, he volunteered the information that "he felt better."

We have operated upon about twenty of these cases, but it is too early to give any report of mental improvement if any, yet in every case the report has come to me by the child or the parents that they are feeling better than before operation, and in many cases the parents have reported marked improvement in temperament. The mother of one of the latest boys told me that he was very stupid and quiet, that the older boys picked on him all the time and set him up to all kinds of mischief, but since the operation, he wants to fight them all. The children don't understand it, but he is evidently beginning to have convictions of his own.

Gradually we are getting the mothers interested. Every mother wants her child to be as bright as the other children, and to have as good a chance, and we are explaining that their children are not having a fair chance. Several during the past week have asked for operation because of improvement in some of the older cases. But, you say, are there no exceptions? Yes, seemingly. The principal of one school was talking to me about this the other day. The pupil in question, a little girl with onesided blindness, reduced hearing and tonsils and adenoids, is exceedingly bright. The principal says, "She has improved steadily in her work during the year. Her teacher notices increasing deafness, and with it increasing eagerness on the part of the child to hear. On the other hand, her brother who is sadly in need of care, is degenerating. During the year his power to concentrate has rapidly diminished. He too is far from well. He is the "tool" for older boys, not seeming to be able to see it. I think it would be quite as worth while to find out why the girl is not affected." Cannot you see that this seeming exception is a very strong argument for the rule? Cannot you see that this girl is using up nervous energy in trying to keep up, which should be used in going ahead?

But someone says, "you throat men are tonsil and adenoid mad." Perhaps so, but so far as I am concerned, I believe so thoroughly in the advisability of removing them, that could I have a monument composed of every tonsil and adenoid in Rochester I would trust to the future for my commendation. And I believe my brother specialists feel the same way.

But let me repeat, every defect, whether it be of the eyes, nose, throat, mouth or gastro-intestinal tract adds its weight to the load which is depressing or retarding the child's mentality.

Every year the number of medical students decreases and the number of drug stores, patent medicines and quack doctors increases simply, I believe, because the physicians will not take the people into their confidence about these matters and explain to them how very much easier it is to keep a child well than it is to cure it after it is sick, how much easier it is to prevent feeble-mindedness than it is to cure the child after his mentality has begun to go.

Cannot we become a unit in this matter which is of such importance to the future of our city? What a monument to our profession! Rochester, not only the home of some of the world's greatest industries, but the home of the best cared for, healthiest, brightest and best lot of children in the country. Isn't it worth working for?

27 ROWLEY STREET.

